# School Interventions for Bullying–Cyberbullying Prevention in Adolescents: Insights from the UPRIGHT and CREEP Projects

**DOI:** 10.3390/ijerph182111697

**Published:** 2021-11-07

**Authors:** Silvia Gabrielli, Silvia Rizzi, Sara Carbone, Enrico Maria Piras

**Affiliations:** Fondazione Bruno Kessler, Digital Health Lab, 38123 Trento, Italy; srizzi@fbk.eu (S.R.); saracarb@gmail.com (S.C.); piras@fbk.eu (E.M.P.)

**Keywords:** bullying, cyberbullying, adolescence, digital interventions, mental health, resilience, co-design

## Abstract

Background: Several challenges and emotional demands characterize adolescence, affecting the mental well-being of youths. Among these, bullying and cyberbullying are recognized nowadays as a major social problem, affecting more than one-third of adolescents, with extensive negative consequences for the victims involved, such as lower self-esteem, increased loneliness, depression, and anxiety. School programs and interventions that foster resilience, coping, and well-being are particularly important during adolescence as protective and preventive factors against the consequences of (cyber)bullying. The paper presents two recent co-designed interventions for (cyber)bullying prevention deployed in Europe, targeting early adolescents and their school communities. Methods: The UPRIGHT project developed an evidence-based, whole-school intervention to train resilience as a protective factor to promote mental well-being in adolescents, in a cross-national perspective. The CREEP project designed and implemented digital interventions to support schools in (i) early detection of cyberbullying events on social media and (ii) coaching adolescents (victims, bullies, bystanders) on how to cope with (cyber)bullying behaviors. Results: The main challenges and insights collected during the design and implementation of both interventions are discussed to inform future research and practice. Conclusion: The feasibility and acceptance of prevention programs are key to the reducing risk of (cyber)bullying and improving the psychological well-being of early adolescents.

## 1. Introduction

Adolescence is characterized by several physical, socio-emotional challenges, affecting the mental well-being of youngsters. Bullying and cyberbullying behaviors represent nowadays a major social problem, affecting 37% of adolescents [1,2], with considerable negative consequences for the victims involved. Traditional bullying can assume a direct form, such as hitting, making threats, and name-calling, or can occur in an indirect form, such as rumor-spreading and social exclusion [3,4]. In contrast to bullying, cyberbullying involves the use of electronic communication devices, with mobile phones (calls and text messages), social media, and instant messaging on the internet being the most frequent platforms for cyberbullying [5,6]. The ability to grant perpetrators anonymity and the ease of dissemination of materials online distinctly differentiate cyberbullying from traditional bullying [7,8]. Adolescent victims of traditional bullying and cyberbullying have a higher susceptibility to somatic (e.g., flu), psychosomatic (e.g., sleeping disorders), and psychological (e.g., anxiety, depression) disorders [9,10,11]. They are also more likely to have a higher risk of self-harm and suicide, poorer internalization skills, poor academic achievement, and school absenteeism [12,13].

### 1.1. Key Factors of Bullying and Cyberbullying Interventions

Several studies have developed anti-bullying and cyberbullying interventions to help curb bullying occurrences. These include cognitive behavioral programs [14], educational programs [15,16,17], and peer support schemes [18]. Among educational programs, the KiVa program is rooted in the belief that a bystander’s behavior is expected to have a direct impact on the behavior of bullies and thus is focused on encouraging bystanders to support and protect victimized peers. Its effectiveness in countering both traditional bullying and cyberbullying has been demonstrated in previous work [16,19,20]. The Second Step program, a skill-based (e.g., problem-solving skills, emotion management, and empathy) educational intervention, was used in two studies to reduce traditional bullying [21,22], with one of them combining the program with an additional cultural awareness training [22]. The ViSC program, originally designed to reduce cyberbullying, was also implemented in two studies [17,23]; however, it was used in conjunction with rational emotive behavioral therapy, and its effectiveness for traditional bullying was investigated [17]. Regarding the effectiveness of these educational programs in terms of the type of approach followed, recent reviews showed that: (i) whole-school-focused interventions were more effective in reducing bullying than interventions delivered through classroom curricula or social skills training alone [24], and (ii) whole-school-based interventions that included a combination of school rules and sanctions, teacher training, classroom activities, and individual counselling were more effective than curricula-based interventions [25].

More recently, UNESCO and the World Anti-Bullying Forum launched a series of initiatives to prevent school bullying by promoting the whole-education approach to help design and deliver evidence-based responses to bullying in and around schools. The whole-education approach ensures that local school initiatives recognize the importance of the interconnectedness of the school with the wider community, including education, technological and societal systems, values, and pressures [26].

Regarding the duration of anti-bullying interventions, previous reviews [27] revealed a direct link between the duration (9 months and above) and intensity (20 h or more) of a program to its effectiveness. This dose-response relationship was also mirrored in previous studies [28,29], in which program effectiveness on traditional bullying was linked to the number of components of a program. Moreover, systematic reviews reported that parent involvement in the program, as well as parent-teacher meetings, were significantly related to decreases in traditional bullying perpetration and victimization. Previous studies also showed that schools can benefit from engaging technology-savvy content experts to conduct training sessions for teachers to better equip these teachers with how to manage cyberbullying more effectively [30].

### 1.2. Resilience as a Protective Factor for Bullying and Cyberbullying

Psychological resilience has been defined as “the capacity to spring back, rebound, successfully adapt in the face of adversity, and develop social and academic competence despite exposure to severe stress…or simply the stress of today’s world” [31]. Resilience should not be intended as an internal characteristic that some individuals have and others lack, but one that can be bolstered by environmental factors, such as programs promoting healthy youth development irrespective of the intensity of the challenging factors or high-risk behaviors adolescents might be facing [32].

Recent studies show that resilience can serve as a protective factor to help youth cope with bullying or cyberbullying [33]. There has been a renewed call among scholars who study bullying that the answer lies less in attenuating risks and dangers, and more in developing protective assets to address social and emotional deficiencies [34,35]. Ideally, resilience-building can occur at a young age, and PATHS (Promoting Alternative Thinking Strategies) is one program that has demonstrated success through randomized controlled trials among elementary schoolers [36,37,38]. Involving over 50 lessons designed to produce social and emotional growth while navigating affect regulation, conflict, and problem-solving, this multi-year program can help to produce the desired changes when implemented with fidelity and the support of all stakeholders within and around a school [39,40]. Along similar lines, the classroom-based programs Steps to Respect and Second Step for elementary and middle schoolers contribute to some indicators of resilience through their focus on social competence and social problem solving [41,42]. Skrzypiec and Wyra [43] recently found in a study involving young adolescents from 11 countries that the role of resilience was as a mediator of well-being. They have suggested that repeated harmful aggression erodes an individual’s resilience, which directly impacts well-being. It seems that certain components of resilience such as levels of optimism, self-efficacy, adaptability, tolerance, and sensitivity decrease the probability of students’ victimization [44]. Therefore, evidence-based research on interventions fostering both the individual and the environmental factors contributing to youth resilience and well-being shows that these assets are more likely to be effective in preventing (cyber)bullying and high-risk behaviors in school and out-of-school settings.

### 1.3. Objective of the Paper

This paper presents the main insights and challenges faced during the design and implementation of two recent European projects: (i) the Horizon 2020 (H2020) UPRIGHT project, which focuses on creating a culture of well-being in middle schools by means of resilience training, following a whole-school approach; and (ii) the EIT Digital CREEP project, which has been designed to deploy innovative digital interventions to support school educators in the early detection and prevention of (cyber)bullying episodes affecting adolescents’ mental well-being and coaching on coping with them.

These insights are key to informing future researchers, educators, and practitioners on how to deploy more effective programs and digital solutions for (cyber)bullying prevention in schools.

## 2. Resilience Training as a Protective Factor of (Cyber)Bullying in Adolescence: The UPRIGHT Project

### 2.1. Overview of the Resilience-Based Intervention to Promote Mental Well-Being of Adolescents

The UPRIGHT research project is a randomized, controlled (two parallel groups) multicenter trial involving nearly 6000 adolescents and their families in five regions, including Spain, Italy, Poland, Denmark, and Iceland (an additional partner in Norway contributed to the intervention and study design). The UPRIGHT project is a research and innovation project funded by the European Union’s Horizon 2020 Research and Innovation program. The program is aimed at promoting a culture that enhances mental well-being in the school community by using a whole-school approach and involves building the resilience of students in the age group 12–14, their families, and school professionals [45].

The methodological approach on which the UPRIGHT program is based has the following main characteristics: co-creation of the intervention, involving the adolescents themselves and the key stakeholders in the school community (e.g., teachers, families, school psychologists…), through participatory actions [46]; collaboration with the territory and adaptation to regional needs; implementation of a training intervention carried out from a systemic perspective; evaluation of effectiveness according to the principles of empirical evidence; use of mixed methodologies to test its effectiveness, acceptability, and cost-effectiveness.

The theoretical framework of the UPRIGHT resilience program was initially based on an extensive review of existing evidence-based resilience training interventions in schools. The board of experts, composed of mental health professionals within the UPRIGHT consortium, defined the theoretical framework of the program by ensuring that the most important skills to be bolstered during adolescence were included, avoiding redundancy, and allowing feasibility of the model implementation in schools [45].

**Figure 1:**
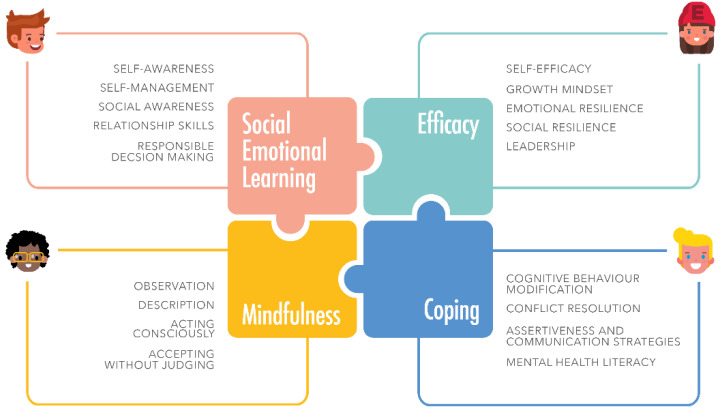
Components and skills of UPRIGHT program.

The final UPRIGHT program comprises four competencies: mindfulness, coping, efficacy, and social and emotional learning, which are promoted through 18 specific skills (Figure 1).

Coping refers to a set of cognitive and behavioral efforts to manage specific external and/or internal demands that are assessed as difficult to cope with in relation to the resources the person feels he or she has [47]. Efficacy is understood as the ability to produce an expected result, is expressed as ‘behavioral performance’, and refers to a person’s perception of their own capabilities [48]. Social Emotional Learning (SEL) is the process by which children and adults acquire and effectively apply knowledge, attitudes, and skills for understanding and managing emotions, setting and achieving positive goals, feeling and demonstrating empathy for others, establishing and maintaining positive relationships, and finally, making responsible decisions [49]. Mindfulness, which is a transversal competency to the previous ones, is defined as the ability to act with full awareness, that is, “knowing what you are doing, while you are doing it” [50], an attitude that comes from intentionally paying attention to the present moment, without judging.

The UPRIGHT intervention works from a systemic perspective by following the whole-school approach [51], i.e., a multi-component approach that aims to include and mobilize the totality of resources in the school context to promote well-being and address any mental health issues.

Teachers benefit from a training program of about 20 h, which provides them with the conceptual framework underlying UPRIGHT, a theoretical framework for each skill, materials to carry out the activities with the students, and experiential training based on the methodologies that the teachers can adopt in the classroom. In the context of the research project, the UPRIGHT team in each pilot site offered group meetings dedicated to the children’s families, with the aim of encouraging and consolidating the climate of collaboration between the school system and the family environment. In addition, special materials were prepared for families, made available through an online web platform (www.uprightprogram.eu, accessed on 6 November 2021), providing three dedicated sections that reflect the ecology of the whole-school approach, i.e., “school”, “family” and “community”.

Schools that committed to participate in the project were stratified according to the number of attending children, location (rural or other), and socioeconomic status. Then, block randomization was performed, and schools were distributed to intervention or control groups.

The UPRIGHT intervention has been implemented over three consecutive school years, from September 2018 until June 2021, in five different pan-European regions (Basque Country in Spain, Trentino region in Italy, Lower Silesia in Poland, Denmark, and Reykjavik area in Iceland) with a total of 34 schools involved, including teachers, and 12 to 14-year-old adolescents and their families.

The intervention consists of two different programs implemented in two consecutive school years: “Well-being for US”, (intensive training, where individual resilience is promoted in 18–24 sessions); and “Well-being for ALL”, when the learning is open to the entire school community to foster collective resilience. During the program “Well-being for US”, all stakeholders are trained in the 18 resilience skills of the UPRIGHT framework. The structure for each session with adolescents consists of: Recall of the previous lesson: what have we learned/applied?; Food for thought: opening questions for the new skill to ignite interest and motivation; Introduction to the selected skill of the lesson; Illustration of the skill (though the use of dilemmas, stories, videos…); Exercises for hands-on experiences with the skills; Mindfulness exercise to create presence and attention; Transfer exercise such as “how can this skill be applied outside this lesson?”.

The “Well-being for ALL” aims to reinforce the effect of the “Well-being for US” training in youths, but it also targets the whole school community to foster collective resilience. Different collective activities were organized at the school level: the displaying of school posters created by the UPRIGHT consortium, checking out the social media accounts, practicing mindfulness during the school year, and implementing four activities (such as writing a gratitude letter, participating in a resilience photo contest, or playing the happiness spin), chosen from a catalogue included in the program manual.

**Figure 2:**
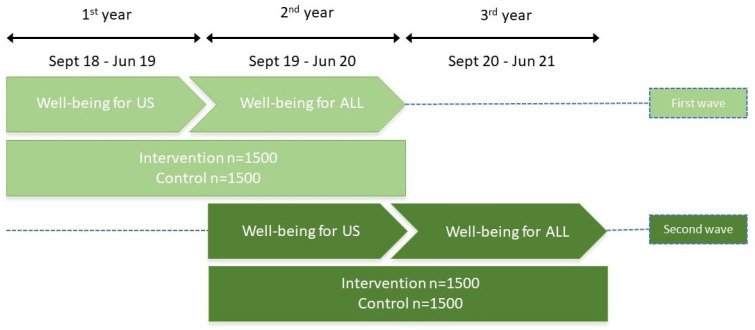
UPRIGHT overall implementation phases.

The UPRIGHT intervention was implemented in all the intervention schools twice (two waves) during the duration of the research project (Figure 2).

The full program will be downloadable at the beginning of 2022 from the UPRIGHT’s project website www.uprightproject.eu (accessed on 6 November 2021) once the evaluation of its effectiveness is completed. In this paper, the focus is on the insights and challenges met during the project implementation in schools, while the findings on the effectiveness of the program on resilience training will be reported in a future project publication.

### 2.2. Insights and Challenges Met in the Project Implementation in Schools

The UPRIGHT implementation brought several challenges which, in turn, provided useful insights for future projects’ implementation.

The project had good geographical coverage, involving 2845 adolescents and 2430 families in the intervention group and 1615 adolescents and 2227 families in the control group, to validate the intervention. Adolescents were 12–14 years old, and 51% were females.

Table 1 reports the rates of bullying and cyberbullying found in the descriptive analysis of the two waves of implementation of UPRIGHT with adolescents (school years 2018–2021), regarding self-reported behaviors of being a victim of bullying or cyberbullying and being a perpetrator of bullying or cyberbullying (considering both control and intervention schools’ adolescents), as measured by employing the eight-item screen used in WHO’s Health Behavior in School-Aged Children survey (HBSC) [52] (e.g., frequency of being bullied or cyber-bullied in the preceding 2 months, and taking part in a bullying or cyber-bullying episode). These data show that bullying and cyberbullying are affecting the schools involved in UPRIGHT, although self-reporting measures may underestimate the real dimension of the phenomenon.

Northern countries (especially Denmark and Iceland) resulted in being more familiar with such training programs, laying also on well-rooted connections among the schools and their local communities, while Southern countries (such as Italy and Spain) required a greater effort in fostering and integrating the well-being culture as a part of the ordinary school curricula. Despite difficulties raised by the COVID-19 impact on the implementation years 2020–2021, all schools were able to align and implement the intervention by adapting the procedures to the restrictions of the pandemic. As an example, schools promoted digital activities compatible with distance learning during the lockdown (e.g., a photo contest on the topic of resilience) or other community activities from the ones offered by UPRIGHT not requiring physical proximity.

The “Well-being for US” program (intensive training) was implemented by pilot sites (Italy, Spain, Denmark, Poland, and Iceland) with some difficulties related to the teachers’ workload. They were asked to attend an UPRIGHT training program of about 20 h and then to deliver the resilience training to their students for approximately 18–24 school hours per year. As shown by previous research [26], longer duration and higher intensity of prevention programs (more than 20 training hours) is key to ensure their effectiveness. As far as the involvement of school staff is concerned, teachers from all schools involved showed high interest and participation in the training: a total number of 408 secondary school teachers were trained, largely exceeding the original target number of 50 (including teachers and school staff). During the qualitative evaluation of the program (based on semi-structured interviews) and the completion of an ad hoc satisfaction questionnaire at the end of the training, teachers reported that they appreciated the training, the project materials, as well as the learning and benefiting in terms of their personal well-being. One challenge they reported in implementing the project was that they felt they lacked expertise in mental health topics, something that can be relevant to the proper deployment of cyberbullying prevention projects [30], and they asked for professional support in delivering training on more technical skills (e.g., mindfulness sessions). A recommendation for the future implementation of prevention programs such as UPRIGHT is to raise as much as possible the number of teachers and school staff trained on the program, as well as to consider a possible differentiation of their roles in the delivery of the training to students, according to their personal motivation, needs, and commitment in such training.

Although schools are typically used to deploy shorter types of interventions (e.g., experts’ workshops, 1-year projects) for prevention, usually delivered by external staff, the UPRIGHT project promoted training delivered directly by the schools’ teachers, since this is likely to be more effective [53], as teachers know how to better tailor sessions to the educational needs of their students. Moreover, teachers spend a great amount of time with students which provides them with multiple opportunities to integrate the resilience training in these interactions, intercalate resilience teaching in the usual academic subject, act as a role model for children in resilience skills, and create a culture of resilience and well-being throughout the school year and not only when external staff comes to the school.

Engaging families during the project represented another challenge, since this group of stakeholders is quite difficult to keep involved, especially when multi-year interventions are deployed. This obstacle was partially overcome in UPRIGHT by offering informative webinars and providing an online platform and materials to facilitate access to the intervention also remotely, including clear instructions, tutorials, and videos. As an example, the UPRIGHT platform was visited and used by about 3700 different users during the three school years (mainly families and teachers/school staff), which shows some interest by families in using this type of support provided by technology for getting informed and trained on prevention programs.

Notwithstanding its impact, COVID-19 also provided an opportunity for experimenting with possible adaptations of the UPRIGHT training contents. This enhanced the accessibility of the program and facilitated the possibility of deploying the training resources produced during the project also in future interventions for mental well-being and cyberbullying prevention.

Flexibility, variety in the training provided, and dynamism in managing the training sessions are key facilitating factors to consider for ensuring the engagement of students in the learning process.

Students showed appreciation for the training methodologies used in UPRIGHT for stories, photos, and videos supporting their social skills training. When asked to define their experience with the UPRIGHT program, during focus groups organized in all pilot sites for the qualitative evaluation of the program (two school classes per pilot site involved after each wave, for a total of 20 classes overall), the words most used were interesting, helpful, and useful for improving self-awareness, emotional regulation, and social awareness. Participants also reported to have experienced a change in their perception of resilience; more specifically, they felt to have improved their skills related to social awareness, assertiveness, and communication strategies, conflict resolution, responsible decision-making, and growth mindset (i.e., being positive about challenges in facing sports challenges and examinations), feeling also more open-minded (e.g., in considering options, others’ opinions, and solutions).

In general, the analysis of the feedback from students participating in the focus groups evidenced a positive effect of the program in the school atmosphere, improving relationships, relaxation, empathy, and tolerance, preventing teasing and conflicts, as well as improving the relationship with their teachers.

## 3. How to Design Technology-Enhanced Interventions to Early Detect and Cope with (Cyber)Bullying: The CREEP Project

### 3.1. Rationale

Cyberbullying requires the use of electronic communication devices to be performed, and it is mostly performed through the most popular applications utilized by teenagers such as social media [5,6]. It is not surprising that several countermeasures to prevent and contrast cyberbullying promote restrictions to their use, education or monitoring of online activities of teenagers. In this respect, early-age or un-scrutinized access to social media, online gaming, or instant messaging platforms are regarded as a potential source of exposure to harassment of a vulnerable population. Such a perspective is in line with the cautionary strategy implied in ‘parental control’, where the web and technologies to access it are portrayed as inherently dangerous for unsupervised minors. In this respect, several programs promote kids/adolescent education and parents/adults’ involvement, or a combination of the two, to mitigate the risks and offer timely support to victims, as reported in Section 1.1.

The CREEP (Cyberbullying Effects Prevention) project (http://creep-project.eu/, accessed on 6 November 2021) stemmed from a critical analysis of such evidence-based approaches, and it aimed at addressing two main issues. Firstly, a parental control approach puts a significant burden on parents, requiring a non-scalable monitoring activity of their children, which is most likely to happen in resourceful families. Numerous or single-parents’ families as well as parents with limited digital or linguistic proficiency (e.g., first-generation migrants) can be excluded or have limited benefits from tools that allow them to perform a monitoring of the online activities of their children. Moreover, since such tools are already offered in the market, there was no need, from a research perspective, to replicate them. Secondly, the project regarded technologies as resources, or ‘digital allies’, thus overturning the established paradigm of the approach to cyberbullying contrast, which represents technologies as threats.

The CREEP project addressed cyberbullying prevention through the design of technologies realized for two different purposes. One technology is “CREEP Semantic Technology”, a social media analytics platform developed to provide schools (or local department of education) with an artificial intelligent hate speech and cyberbullying detection system. The platform allows to monitor the online textual utterances exchanged in the comments of open profiles of social media applications (i.e., Instagram), detect potential cyberbullying interaction, cluster the network of relevant interaction, anonymize the sources, and report to a school manager in charge of the fine-grained analysis of the cases reported by the system. CREEP Semantic Technology is a cyberbullying detection tool whose aim is to provide managers with an overview of emerging threats in their schools. Through its use, for instance, school managers can be alerted when a (configurable) threshold of abusive language is detected on social media, providing them with a tool that helps them to classify the prevailing cyberbullying category (e.g., body shaming, hate speech, sexism, racism), thus allowing for an informed decision regarding the most appropriate countermeasures. The other technology is the “CREEP Virtual Coach”, a mobile application developed to provide teenagers with digital support to cope with, report, and prevent cyberbullying [54]. CREEP Virtual Coach profiles teenagers through a questionnaire designed to gather information on cyberbullying acts experienced or witnessed, thus providing teenagers with recommendations and psycho-educational materials (in the format of short video-cartoons) to strengthen their resilience and coping abilities.

A description of the technical details of the CREEP features is out of the scope of this paper (see [55] for an overview). In the following section, we discuss some of the main challenges associated with the design of such digital interventions and the lessons learned in the process.

### 3.2. Design and Methodology Deployed in the Digital Interventions

Artificial Intelligence (AI) refers to the computational ability to perform tasks normally associated with human intelligence and discernment, such as speech recognition, decision-making or decision support, and pattern recognition. There are several strategies to create AI-based systems, most of them requiring data sets to train the system to act in a way that resembles human behavior. While humans learn also by virtue of analogy and imitation, AI training needs a significant amount of data to be able to detect patterns. The challenge in designing AI to support cyberbullying-related prevention activities is twofold. On the one hand, harassment can be elusive also for humans to detect, it does not follow strict rules, and its interpretation can vary across time and social groups. On the other hand, accessing for research purposes datasets of actual cyberbullying interactions, which are criminal activities according to the law, poses legal and ethical issues. Cyberbullying dynamics have often been explored through either datasets voluntarily ‘donated’ by users [56] or corpora scraped from social media platforms [57]. The main limitation of such research strategies is that they require large datasets from which to extract a restricted number of harassing interactions, and that each dataset is language-specific.

With these limitations in mind, the CREEP project adopted a different strategy to create cyberbullying corpora, namely a participatory laboratorial activity called “CREEPY Roleplaying”. Seven secondary schools (three lower; four upper) in four Italian regions (Trento and Turin in northern Italy, Palermo in Sicily, and Nice in Cote d’Azur, France) were involved in four workshops, with each class organized across four to five weeks. The workshops were conducted by a mixed team of computational linguists and sociologists and around this structure:

– First meeting: Participatory lecturing—Discussing students’ experiences and opinions regarding cyberbullying.
– Second meeting: Annotation of interactions—Students in pairs annotate threads gathered from Instagram and Twitter, categorizing them according to hate speech categories.
– Third meeting: Introduction to Roleplaying—Students are assigned a role and play a cyberbullying simulation.
– Fourth meeting: Participatory analysis—Researchers present the preliminary analysis of the experimentation and elicit students’ interpretation.

The core of the workshop is the roleplaying [58,59], in which students participate in an immersive simulation some hours each day for three to four days on an instant messaging platform (i.e., WhatsApp). The purpose of the simulation is to collect realistic data on cyberbullying interactions while shielding participants from the harmful effects of a relationship that may take on even violent overtones. To this aim, strict measures were put in place to avoid the simulation becoming personal and ensure constant vigilance on the process.

Parents’ authorization to the workshop was required beforehand. Students could opt out (even if parents allowed their participation) before the roleplaying or any time they felt uneasy about it. The simulation started with a scenario crafted by a researcher which presented a realistic, but not real, situation. Researchers developed different scenarios for each class age involved. Each scenario was modeled around a ‘trigger’ which could have led to initiate a cyberbullying interaction (e.g., photographs shared without the permission of the person depicted, revealing sexual preferences, snitching on someone with teachers). Hints regarding the details of scenarios were gathered in the first meeting (participatory lecturing), where students discussed lived experience of online harassment. Teachers and parents were involved to refine scenarios to avoid teenagers reliving traumatic personal experiences. Students were assigned a role (bully, bystander, victim) and a nickname. Every two days, the scenario and roles were changed to ensure each participant played all roles. Students were given a set of ten rules to follow (e.g., stick to the simulation and offend the ‘persona’, not your classmate): one researcher and one teacher were always in the group where the simulation took place and could exclude participants who did not adhere.

The CREEPY Roleplaying permitted to gather a large corpus of simulated cyberbullying interactions to be analyzed [60] and train CREEP Semantic Technology and the CREEP Virtual Coach. The preliminary screening allowed us to select some portions of the dataset and conduct a participatory analysis with the students involved to determine the realism of the data gathered. Moreover, the participatory analysis at school allowed to address the cyberbullying phenomenon from the point of view of the students, favoring a user-centric perspective, and discussing the co-creation of strategies whilst avoiding top-down approaches.

### 3.3. Insights and Lessons Learned from the Project Deployment in Schools

The digital interventions developed by the CREEP project in their prototypical implementations were positively evaluated by the participants involved in their assessment, as reported in [54]. Both the workshops and the digital tools were also evaluated by the participants through an ad hoc questionnaire. The dimensions explored in the survey were: perceived usefulness, engagement, interest, and adequacy of the interventions. A detailed description of the results is out of the scope of this paper. In short, students appreciated the CREEP Virtual Coach both for its ease of use and for the psycho-educational materials offered. The evaluation helped to assess the feasibility of the approach deployed and aimed at providing support directly to teenagers rather than delivering it through their parents [54]. Much research confirmed that only a minority of teenagers perceive their parents or other adults as the primary source of help in cases of cyberbullying, preferring to manage it by themselves or ask for peer support. As age increases, this tendency grows until parents are given a very marginal role in the management of aggressions suffered on the Internet. This behavior is consistent with the pathway to autonomy, and it reinforces the benefits of digital interventions that can accompany the teenager from the inception of their online activities and provides a first line of support when needed, complemented by monitoring tools for parents that are widely available on the most popular app stores.

A demo version of the CREEP Semantic Technology was presented to teachers and school managers (the fully integrated technology was deployed at the very end of the project cycle). In 2017, the Italian parliament approved a Cyberbullying bill which envisioned new responsibilities for the schools, but online activities of students are largely unknown to teachers. Teachers and managers appreciated the possibility of having a tool at their disposal to capture early signs of the surge of cyberbullying or hate speech among their students.

CREEPY Roleplaying was created as an instrumental response to a research challenge. Over the course of the two-years’ project, it became a methodology in its own right, and several other schools required it as a ‘professional service’ to sensitize adolescents to the phenomena. The experiential learning stimulated by the roleplaying activities remains non-scalable, and it is a resource-intensive approach to foster an in-depth understanding of the phenomena.

The core partners of the CREEP consortium are currently advancing, formalizing, and validating the digital tools and methodologies developed through Kid_Actions (https://www.kidactions.eu/, accessed on 6 November 2021), a two-year EU project (REC Action Grant) in partnership with NGOs involved in education and human rights promotion. The Kid_Action project will allow us to refine and test the digital tools developed in CREEP by conducting workshops in several European countries.

## 4. Conclusions

This paper has presented the main insights and challenges met in the design and implementation of two European projects aimed at supporting schools with the training of resilience as a protective factor to prevent (cyber)bullying and with the deployment of innovative digital interventions to detect and prevent this phenomenon early.

Starting from the analysis of evidence-based interventions and approaches deployed so far to promote mental well-being of adolescents, both UPRIGHT and CREEP have demonstrated the feasibility and raising interest of schools for the co-design and adoption of such interventions, which has become particularly relevant in the time of the COVID-19 pandemic.

The whole-school approach and the participatory design methods deployed in the implementation of these programs and interventions have helped us to detect and react early to possible obstacles that school communities might find in their adoption, facilitating a more structured and long-term development of school policies to promote a real culture of mental well-being in their communities.

The insights presented can be relevant to inform future research and practice on the role of resilience training and digital interventions to address the risk of (cyber)bullying from a prevention perspective at school. In this view, the UPRIGHT project has recently been selected and included on the School Education Gateway website (https://www.schooleducationgateway.eu/en/pub/resources/toolkitsforschools/detail.cfm?n=21864, accessed on 6 November 2021) as part of the European Toolkit for Schools.

## 5. Limitations and Further Developments

This paper contributes to the state-of-the-art knowledge on evidence-based interventions for (cyber)bullying prevention and the promotion of adolescents’ well-being. However, more research is needed to fully assess the effectiveness of resilience training and digital interventions as protective factors able to reduce high-risk behaviors, such as (cyber)bullying, in school and out of school environments.

Future research may leverage on the mediator effect of resilience training, deployed in presence but also with the support of digital interventions, to better engage adolescents and their communities in the development of life-skills, thus favoring mental well-being and the achievement of the intended educational goals.

## Figures and Tables

**Table 1:** Rates of bullying and cyberbullying self-reported by UPRIGHT adolescents.

	N (%)
VICTIM of Bullying	428 (14.9)
VICTIM of CyberBullying	254 (9)
PERPETRATOR of Bullying	305 (10.6)
PERPETRATOR of CyberBullying	198 (6.9)

## Data Availability

Not applicable.

## References

[1] Patchin J.W., Cyberbullying Research Center (2019). 2019 Cyberbullying Data. https://cyberbullying.org/2019-cyberbullying-data.

[2] Craig W., Harel-Fisch Y., Fogel-Grinvald H., Dostaler S., Hetland J., Simons-Morton B., Molcho M., de Mato M.G., Overpeck M., Due P. (2009). A cross-national profile of bullying and victimization among adolescents in 40 countries. Int. J. Public Health.

[3] Bjorkqvist K., Lagerspetz K.M.J., Kaukiainen A. (1992). Do girls manipulate and boys fight? Developmental trends in regard to direct and indirect aggression. Aggress. Behav..

[4] Ericson N. (2001). Addressing the Problem of Juvenile Bullying.

[5] Kowalski R.M., Giumetti G.W., Schroeder A.N., Lattanner M.R. (2014). Bullying in the digital age: A critical review and meta- analysis of cyberbullying research among youth. Psychol. Bull..

[6] Smith P.K., Mahdavi J., Carvalho M., Fisher S., Russell S., Tippett N. (2008). Cyberbullying: Its nature and impact in secondary school pupils. J. Child. Psychol. Psychiatry.

[7] Cross D., Lester L., Barnes A. (2015). A longitudinal study of the social and emotional predictors and consequences of cyber and tra- ditional bullying victimisation. Int. J. Public Health.

[8] Zuckerman D. (2016). Bullying harms victims and perpetrators of all ages. Health Prog. (St. Louis Mo.).

[9] Beckman L., Hagquist C., Hellström L. (2012). Does the association with psychosomatic health problems differ between cyberbullying and traditional bullying?. Emot. Behav. Difficulties.

[10] Gini G., Pozzoli T. (2013). Bullied children and psychosomatic problems: A meta-analysis. Pediatrics.

[11] Schneider S.K., Donnell L., Stueve A., Coulter R.W.S. (2012). Cyberbullying, school bullying, and psychological distress: A regional census of high school students. Am. J. Public Health.

[12] Kumpulainen K., Rasanen E. (2000). Children involved in bullying at elementary school age: Their psychiatric symptoms and deviance in adolescence: An epidemiological sample. Child. Abus. Neglect..

[13] Wolke D., Lereya S.T. (2015). Long-term effects of bullying. Arch. Dis. Child..

[14] Gokkaya F. (2017). Peer bullying in schools: A cognitive behavioral intervention program. Child. Adolesc. Ment. Health.

[15] Limber S.P. (2003). Efforts to address bullying in U.S. schools. Am. J. Health Educ..

[16] Nocentini A., Menesini E. (2016). KiVa anti-bullying program in Italy: Evidence of effectiveness in a randomized control trial. Prev. Sci..

[17] Trip S., Bora C., Sipos-Gug S., Tocai I., Gradinger P., Yanagida T., Strohmeier D. (2015). Bullying prevention in schools by targeting cognitions, emotions, and behavior: Evaluating the effectiveness of the REBE-ViSC program. J. Couns. Psychol..

[18] Tzani-Pepelasi C., Ioannou M., Synnott J., McDonnell D. (2019). Peer support at schools: The buddy approach as a prevention and intervention strategy for school bullying. Int. J. Bullying Prev..

[19] Karna A., Voeten M.J.M., Little T.D., Alanen E., Poskiparta E.H., Salmivalli C. (2013). Effectiveness of the KiVa antibullying program: Grades 1–3 and 7–9. J. Educ. Psychol..

[20] Williford A., Elledge L.C., Boulton A.J., DePaolis K.J., Little T.D., Salmivalli C. (2013). Effects of the KiVa antibullying program on cyberbullying and cybervictimization frequency among Finnish youth. J. Clin. Child. Adolesc. Psychol..

[21] Espelage D.L., Low S., Polanin J.R., Brown E.C. (2013). The impact of a middle school program to reduce aggression, victimization, and sexual violence. J. Adolesc. Health.

[22] Polanin M.K. (2014). Effects of Cultural Awareness Training in Conjunction with an Established Bullying Prevention Program (3624107). Ph.D. Thesis.

[23] Gradinger P., Yanagida T., Strohmeier D., Spiel C. (2015). Prevention of cyberbullying and cyber victimization: Evaluation of the ViSC Social Competence Program. J. Sch. Violence.

[24] Cantone E., Piras A.P., Vellante M., Preti A., Daníelsdóttir S., D’Aloja E., Lesinskiene S., Angermeyer M.C., Carta M.G., Bhugra D. (2015). Interventions on bullying and cyberbullying in schools: A systematic review. Clin. Pract. Epidemiol. Ment. Health CP EMH.

[25] Shackelton N., Jamal F., Viner R.M., Dickson K., Patton G.C., Bonell C. (2016). School-Level Interventions to Promote Adolescent Health: Systematic Review of Reviews. J. Adolesc. Health.

[26] (2021). UNESCO and World Anti-Bullying Forum. https://en.unesco.org/news/new-series-international-meetings-school-bullying-rolled-out-2021.

[27] Gaffney H., Farrington D.P., Espelage D.L., Ttofi M.M. (2019). Are cyberbullying intervention and prevention programs effective? A systematic and meta-analytical review. Aggress. Violent Behav..

[28] Olweus D. (2005). A useful evaluation design, and effects of the Olweus Bullying Prevention Program. Psychol. Crime Law.

[29] Smith P.K. (1997). Bullying in schools: The UK experience and the Sheffield Anti-Bullying project. Ir. J. Psychol..

[30] Ng E.D., Chua J.Y.X., Shorey S. (2020). The effectiveness of educational interventions on traditional bullying and cyberbullying among adolescents: A systematic review and meta-analysis. Trauma Violence Abus..

[31] Henderson N., Milstein M.M. (2003). Resiliency in Schools: Making it Happen for Students and Educators.

[32] Herrman H., Stewart D.E., Diaz-Granados N., Berger E.L., Jackson B., Yuen T. (2011). What is resilience?. Can. J. Psychiatry.

[33] Hinduja S., Patchin J.W. (2017). Cultivating youth resilience to prevent bullying and cyberbullying victimization. Child. Abus. Negl..

[34] Gibson J.E., Polad S., Flaspohler P.D., Watts V. (2016). Social emotional learning and bullying prevention. Contemp. Perspect. Res. Bullying Victim. Early Child. Educ..

[35] Low S., Smolkowski K., Cook C. (2016). What constitutes high-quality implementation of SEL programs? A latent class analysis of second Step^®^ implementation. Prev. Sci..

[36] Bierman K.L., Coie J.D., Dodge K.A., Greenberg M.T., Lochman J.E., McMahon R.J., Pinderhughes E. (2010). The effects of a multiyear universal social-emotional learning program: The role of student and school characteristics. J. Consult. Clin. Psychol..

[37] Domitrovich C.E., Cortes R.C., Greenberg M.T. (2007). Improving young children’s social and emotional competence: A randomized trial of the preschool PATHS curriculum. J. Prim. Prev..

[38] Zins J.E. (2004). Building Academic Success on Social and Emotional Learning: What does the Research Say?.

[39] Cooke M.B., Ford J., Levine J., Bourke C., Newell L., Lapidus G. (2007). The effects of city-wide implementation of Second Step on elementary school students’ prosocial and aggressive behaviors. J. Prim. Prev..

[40] Winslow E.B., Sandler I.N., Wolchik S.A. (2005). Building resilience in all children. Handbook of Resilience in Children.

[41] Taub J. (2002). Evaluation of the Second Step violence prevention program at a rural elementary school. Sch. Psychol. Rev..

[42] Taub J., Pearrow M. (2005). Resilience through violence prevention in schools. Handb. Resil. Child..

[43] Skrzypiec G., Wyra M., Skrzypiec G., Wyra M., Didaskalou E. (2019). Global results of peer aggression and well-being study. A Global Perspective of Young Adolescents’ Peer Aggression and Well-Being: Beyond Bullying.

[44] Moore B., Woodcock S. (2017). Resilience to bullying: Towards an alternative to the anti-bullying approach. Educ. Psychol. Pract..

[45] Las Hayas C., Izco-Basurko I., Fullaondo A., Gabrielli S., Zwiefka A., De Manuel Keenoy E. (2019). Upright, a resilience-based intervention to promote mental well-being in schools: Study rationale and methodology for a European randomized controlled trial. BMC Public Health.

[46] Morote R., Las Hayas C., Izco-Basurko I., Anyan F., Fullaondo A., Hjemdal O. (2020). Co-creation and regional adaptation of a resilience-based universal whole-school program in five European regions. Eur. Educ. Res. J..

[47] Lazarus R.S., Folkman S. (1984). Stress, Appraisal and Coping.

[48] N., Sam M.S EFFICACY, in PsychologyDictionary.org, 7 April 2013. https://psychologydictionary.org/efficacy/.

[49] Collaborative for Academic, Social, and Emotional Learning—CASEL. https://casel.org/.

[50] Kabat-Zinn J. (1994). Wherever You Go, There You Are: Mindfulness Meditation in Everyday Life.

[51] Weare K. (2000). Promoting Mental, Rmotional and Social Health: A Whole School Approach.

[52] Currie C., Inchley J., Molcho M., Lenzi M., Veselska Z., Wild F. (2014). Health Behaviour in School-Aged Children (HBSC) Study Protocol: Background, Methodology and Mandatory Items for the 2013/14 Survey.

[53] Fenwick-Smith A., Dahlberg E.E., Thompson S.C. (2018). Systematic review of resilience-enhancing, universal, primary school-based mental health promotion programs. BMC Psychol..

[54] Gabrielli S., Rizzi S., Carbone S., Donisi V. (2020). A Chatbot-Based Coaching Intervention for Adolescents to Promote Life Skills: Pilot Study. JMIR Hum. Factors.

[55] Menini S., Moretti G., Corazza M., Cabrio E., Tonelli S., Villata S. A system to monitor cyberbullying based on message classification and social network analysis. Proceedings of the Third Workshop on Abusive Language Online.

[56] Verheijen L., Spooren W. (2017). The impact of WhatsApp on Dutch youths’ school writing. Media Corpora Humanit..

[57] Van Hee C., Jacobs G., Emmery C., Desmet B., Lefever E., Verhoeven B., Hoste V. (2018). Automatic detection of cyberbullying in social media text. PLoS ONE.

[58] Jones S. (2007). Adding Value to Online Role Plays: Virtual Situated Learning Environment. https://www.researchgate.net/profile/Sandra-Jones-16/publication/228377336_Adding_value_to_online_role_plays_Virtual_situated_learning_environments/links/02e7e520c88efdb0a6000000/Adding-value-to-online-role-plays-Virtual-situated-learning-environments.pdf.

[59] Mooradian J. (2008). Using simulated sessions to enhance clinical social work education. J. Soc. Work. Educ..

[60] Sprugnoli R., Menini S., Tonelli S., Oncini F., Piras E. Creating a whatsapp dataset to study pre-teen cyberbullying. Proceedings of the 2nd Workshop on Abusive Language Online (ALW2).

